# Ferrous Iron Binding Key to Mms6 Magnetite Biomineralisation: A Mechanistic Study to Understand Magnetite Formation Using pH Titration and NMR Spectroscopy

**DOI:** 10.1002/chem.201600322

**Published:** 2016-04-26

**Authors:** Andrea E. Rawlings, Jonathan P. Bramble, Andrea M. Hounslow, Michael P. Williamson, Amy E. Monnington, David J. Cooke, Sarah S. Staniland

**Affiliations:** ^1^ Department of ChemistryUniversity of SheffieldSheffieldS3 7HFUK; ^2^ Department of Molecular Biology and BiotechnologyUniversity of Sheffield, Firth Court, Western BankSheffieldS10 2TNUK; ^3^ Department of Chemical and Biological SciencesUniversity of Huddersfield, QueensgateHuddersfieldHD1 3DHUK

**Keywords:** biomimetic synthesis, magnetite, magnetotactic bacteria, mineralization, NMR spectroscopy

## Abstract

Formation of magnetite nanocrystals by magnetotactic bacteria is controlled by specific proteins which regulate the particles’ nucleation and growth. One such protein is Mms6. This small, amphiphilic protein can self‐assemble and bind ferric ions to aid in magnetite formation. To understand the role of Mms6 during in vitro iron oxide precipitation we have performed in situ pH titrations. We find Mms6 has little effect during ferric salt precipitation, but exerts greatest influence during the incorporation of ferrous ions and conversion of this salt to mixed‐valence iron minerals, suggesting Mms6 has a hitherto unrecorded ferrous iron interacting property which promotes the formation of magnetite in ferrous‐rich solutions. We show ferrous binding to the DEEVE motif within the C‐terminal region of Mms6 by NMR spectroscopy, and model these binding events using molecular simulations. We conclude that Mms6 functions as a magnetite nucleating protein under conditions where ferrous ions predominate.

## Introduction

Essential to many organisms, iron is an important component of many biological processes.^1^ Due to the inherent redox activity and pH sensitivity of this transition metal its presence in cells must be carefully controlled to prevent potentially harmful effects from reactive oxygen species1c or iron precipitation.^2^ Many proteins have therefore evolved to utilise, control and harness the useful capabilities of iron whilst minimizing any potentially damaging side effects.1a, 1b In the case of magnetotactic bacteria (MTB), they have evolved to take advantage of the magnetic characteristics of certain iron oxides by producing biogenic magnetic nanoparticles^3^ within internal lipid vesicles termed magnetosomes.^4^ These vesicles are in effect a nanoreactor for the precise synthesis of, most commonly, the iron oxide magnetite (Fe_3_O_4_).3c, 5 The formation of nanocrystalline magnetite is tightly controlled by a suite of biomineralisation proteins which are present within the lipid membrane of the magnetosome.^6^ The nucleation, crystal growth and regulation of the final size and shape of the particle are strictly regulated by these proteins. A single strain of MTB harbours a highly uniform population of nanoparticles; homogeneous in terms of size, shape, and chemical composition. Research is currently focusing on identifying and characterising these biomineralisation Mms (magnetosome membrane specific) proteins in order to elucidate a detailed understanding of iron oxide biomineralisation.

**Figure 1:**
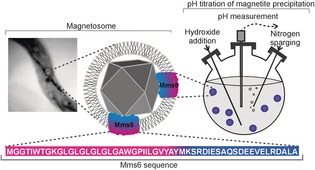
Mms6 in magnetotactic bacterial magnetosome. In blue is the hydrophilic acid‐rich region and in pink is the hydrophobic membrane region with the sequence below. Experimental schematic showing pH recording during addition of base to ferric/ferrous solution with or without Mms6 micelles is also illustrated.

One key protein found tightly bound to the magnetite particles of *Magnetospirillum magneticum* AMB‐1^7^ is a 6 kDa protein, Mms6. Mms6 comprises a hydrophobic N‐terminal region and a hydrophilic C‐terminal region (KSRDIESAQSDEEVELRDALA) which contains a high number of residues with acidic sidechains that have been implicated in the ferric iron binding capability of the protein (Figure 1).^7^, ^8^ If the *mms6* gene is removed from MTB (so Mms6 protein is not produced) the resulting nanoparticles which form are both small and poorly formed compared to the wild‐type.^9^ Mms6 has therefore been classified as an important member of the magnetite biomineralisation mechanism. Due to its amphiphilic sequence, purified Mms6 self‐assembles into micellar structures.8a These large aggregates are able to both bind and accumulate ferric ions from solution,^7^, ^8^, ^10^ and purified assemblies of Mms6 on a biomimetic magnetosome interior surface demonstrate magnetite formation properties, indicating that Mms6 can act as a potential iron oxide nucleation site for subsequent crystal formation.^11^ In addition, the acid rich C‐terminal part of Mms6 has been studied and was found to exhibit some similar characteristics to the full‐length protein such as iron binding and a limited ability to affect magnetite crystal growth.8a, 10, 12

Magnetic nanoparticles have received much attention due to their potential use in a wide range of different applications spanning various scientific disciplines and technologies.^13^ These range from the magnetic components of ferrofluids^14^ and data‐storage devices, to precision applications in medical diagnostics and therapies.^15^ For this latter use, magnetite is one of the most desirable materials due to its magnetic properties and general low toxicity.15a There are a number of synthetic routes for magnetite nanoparticles including thermal decomposition, and high temperature methods in the presence of organic surfactants.^16^ An alternative and simple method of producing magnetite nanoparticles is by room temperature co‐precipitation (RTCP). This results in particles with a high size polydispersity making them unsuitable for critical biomedical applications. Taking inspiration from nature where MTB synthesize magnetite nanoparticles with a high degree of control over size and shape, purified Mms6 has been introduced into RTCP and other reactions.^7^, ^17^ The resulting products demonstrate improved homogeneity in both size and mineral type, suggesting that Mms6 is able to control the formation of magnetite nanoparticles in vitro.^7^, ^17^, ^18^ However, the exact mechanism by which Mms6 achieves this type of control both in vivo and in vitro remains poorly understood.

In this study we analyse the effect of Mms6 during a simple RTCP of magnetite by monitoring differences in the pH profile during the reaction process. This was performed under a range of different reaction conditions to build up a rigorous and detailed picture of Mms6 activity at different ratios of ferric and ferrous iron. A previous study of iron oxide formation using this approach^19^ has provided a methodology by which to investigate the effect of these additives on the crystallisation process. We find that Mms6 exerts its greatest influence over the process in the pH range where ferrous ions are precipitated out of solution (>pH 7). Previous studies have shown that Mms6 interacts with ferric iron but there is, to the best of our knowledge, currently no analysis of any potential interaction with ferrous iron. Therefore, to complement our study we have used 2D NMR spectroscopy to investigate the ferrous‐binding activity of the C‐terminal peptide region of Mms6, which reveals that the peptide has specific interactions with ferrous iron centred around the DEEVE acidic residue cluster. Further insight is gained through molecular dynamics simulations of this cluster and its interactions with both ferric and ferrous iron. This study therefore provides a new perspective on the activity of the Mms6 protein by suggesting it is interacting with both ferric and ferrous iron during the magnetite crystallisation process, and that it is the predominant and specific interaction with ferrous iron that is the key to driving the reaction towards magnetite. This finding has implications for our understanding of magnetite biomineralisation and how we can control magnetite formation in biomimetic magnetic nanoparticle synthesis for wider nanotechnology applications.

## Results

### Room temperature co‐precipitation of magnetite (RTCP)

The precipitation of iron oxides from solution is complex^20^ and proceeds through a number of intermediate iron oxides. A comprehensive study of the co‐precipitation of ferric and ferrous salts by Ruby et al.^19^, ^21^ employed careful pH titration measurements to identify processes occurring during precipitation reactions. The quantities of Mms6 protein available mean we must use much lower concentrations and reaction volumes than those used by Ruby et al. which makes the experiment very sensitive. In this study we use a similar titration method to monitor the progress of the reaction and the nature of the intermediates formed both with and without the addition of Mms6. This allows us to understand if, and how, the protein affects the co‐precipitation process and to explain how Mms6 is able to exert control over the size and the mineral species formed, specifically magnetite, when added to a RTCP reaction.^12^

Here, a solution of mixed valence iron salts is precipitated by slowly raising the pH with the addition of base. There are a number of reactions present in this system19b that can lead to several iron oxide and iron oxyhydroxide products or intermediates, namely the mixed ferric/ferrous minerals magnetite (Fe^2+^Fe^3+^_2_O_4_) [Eq. (3)] and green rusts ([Fe^2+^_4_Fe^3+^_2_(OH)_12_][SO_4_]⋅*x* H_2_O [Eq. (2)] as well as the pure ferrous mineral ferrous hydroxide (Fe^2+^(OH)_2_) [Eq. (1)] and pure ferric minerals schwertmannite ([Fe^3+^_8_O_8_(OH)_6_] [SO_4_]⋅*x* H_2_O [Eq. (5)] and goethite (Fe^3+^O(OH)) [Eq. (4)] at the extremes of the oxidation states, see Equations (1)–(5), from pure ferrous to pure ferric minerals:

(1) $$\mathrm{Fe}{}^{2+}+2\;\mathrm{OH}{}^{-}\to \mathrm{Fe}\left(\mathrm{OH}\right){}_{2}$$

(2) $$4\;\mathrm{Fe}{}^{2+}+2\;\mathrm{Fe}{}^{3+}+12\;\mathrm{OH}{}^{-}+\mathrm{SO}{}_{4}{}^{2-}\to \mathrm{Fe}{}^{\mathrm{II}}{}_{4}\mathrm{Fe}{}^{\mathrm{III}}{}_{2}\left(\mathrm{OH}\right){}_{12}\mathrm{SO}{}_{4}$$

(3) $$\mathrm{Fe}{}^{2+}+2\;\mathrm{Fe}{}^{3+}+8\;\mathrm{OH}{}^{-}\to \mathrm{Fe}{}^{\mathrm{II}}\mathrm{Fe}{}^{\mathrm{III}}{}_{2}O{}_{4}+4\;H{}_{2}O$$

(4) $$\mathrm{Fe}{}^{3+}+3\;\mathrm{OH}{}^{-}\to \mathrm{FeOOH}+2\;H{}_{2}O$$

(5) $$\mathrm{Fe}{}^{3+}+\left[3-2\;z\right]\mathrm{OH}{}^{-}+z\;\mathrm{SO}{}_{4}{}^{2-}\to \mathrm{FeO}\left(\mathrm{OH}\right){}_{\left[1-2\;z\right]}\left(\mathrm{SO}{}_{4}\right){}_{z}2\left[1-z\right]H{}_{2}O$$

**Figure 2:**
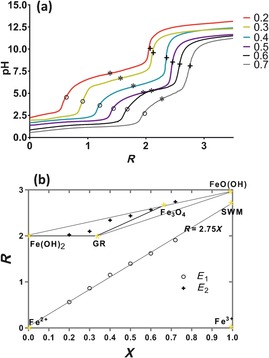
a) pH measurements during room temperature co‐precipitation for total iron concentrations of 50 mm at a rate of 0.05 *R* min^−1^ with equivalence points highlighted by circles (*E*_1_) and crosses (*E*_2_). The central equivalence point is marked by an asterisk (E*). These data are similar to results obtained at much higher concentrations.19a b) Mass balance diagram for the formation of various iron oxides showing the equivalence points from (a). The error was too small to be shown (*E*_1_=zero (to 3 decimal points) and *E*_2_=±1.3 %). GR=green rust and SWM=schwertmannite.

Figure 2 a shows titration curves for the co‐precipitation of ferric and ferrous iron sulfates with NaOH in the absence of Mms6. The molar ratio of Fe^3+^ to total iron is represented by *X* (e.g., Fe^3+^/Fe^2+^ ratio of 1:2 gives *X*=0.33) and ranges from 0 to 1. *R* is the molar ratio between the concentration of NaOH and total iron. For these experiments an effective rate of 0.05 *R* min^−1^ was used for the addition of base, meaning that in our experiments *R* increases linearly with time.2

The step titration curves shown in Figure 2 have three plateaus separated by two steps defined by the equivalence points *E*_1_ (step between plateaus 1 and 2, shown by circles in Figure 2) and *E*_2_ (step between plateaus 2 and 3, shown by crosses in Figure 2). E* is defined as the central point of the second plateau (shown by an asterisk in Figure 2 a).

At the lowest pH conditions the first plateau describes OH^−^ consumption through precipitation of ferric basic salts [e.g., the ferric oxyhydroxide, schwertmannite; Eq. (5)] due to the lower solubility of ferric ions in the presence of base. The middle plateau corresponds to the conversion to, and formation of, a range of possible iron minerals: ferrous hydroxide, green rust, magnetite and goethite [Eqs. (1)–(4)] in various proportions, increasing in amounts of the latter minerals as the ratio of ferric to ferrous ions increases (Figure 2). Interestingly, the ferric oxyhydroxide begins to convert into green rust and magnetite by incorporation of ferrous ions and through electron transfer. Once all the ferric solids have been converted, the excess ferrous ions precipitate as ferrous hydroxide.19b These mixed oxides are retained up to the end of the titration at pH 12.5. At this point the reaction mixture has a highly negative redox potential (approximately −750 mV; see Supporting Information Figure S1) demonstrating its high propensity to oxidise. If left to age, overnight, with a small amount of oxidation, mineral dehydration can occur, converting other iron oxides such as green rust (which can be considered as intermediates) to magnetite. The dark green solution becomes black.

Using the methodology and nomenclature of Ruby et al.19a we plotted the positions of equivalence points derived from the pH titration data on a mass‐balance diagram of *R* versus *X* (Figure 2 b). The first equivalence points at *E*_1_ lie along the line *R*_1_=2.75 *X*. This represents the theoretical relationship for the formation of the sulfated ferric oxyhydroxide schwertmannite, in the first plateau [see Eq. (5), balanced for *z*=1/8, thus OH/Fe ratio=2.75:1].19a The relative quantity of base (*R*) required is linearly dependent on the ratio of ferric/ferrous species (increasing as Fe^3+^ increases; Figure 2 a and b) due to the fact that there are increasing quantities of ferric iron to precipitate. The second equivalence point, *E*_2_, describes the nature and proportions of insoluble iron oxides that are formed in the second plateau (depending on the initial *X* ratio). As discussed above, this could be a mixture of green rusts, magnetite and other ferrous/ferric hydroxide nanoparticles (Supporting Information Figure S2 shows products of the reaction at *X*=0.3 and 0.5 showing a combination of green rust and magnetite at lower *X* ratio, and more pure magnetite at the higher). Stoichiometric magnetite would be formed at *X*=0.67 (i.e., 2 Fe^3+^/1 Fe^2+^). However, it should be noted that extracting a vertical line from the initial *X* ratio to the products is a simplistic representation of the reaction. This reaction has a maturation step after the titration where a small amount of oxidation occurs slowly, overnight, to form the most stable products, resulting in a non‐vertical conversion to the most stable products after this *E*_2_ point. In practice, many magnetite nanoparticle synthesis schemes report higher starting ratios of Fe^2+^ with 0.33≤*X*≤0.6667, in recognition of partial oxidation in the final stages of the process.^7^, ^17^ Generally, increasing the amount of Fe^2+^ in solution results in lower quantities of magnetite and higher quantities of ferrous (oxy)hydroxide particles, while too high a quantity of Fe^3+^ will result in ferric (oxy)hydroxide such as goethite. In summary, the titrations shown in Figure 2 demonstrate the expected pH‐dependent formation of ferrous hydroxide, green rusts and magnetite, as well as the information to understand how and when these are forming and in what quantities.

### Influence of Mms6 on ferrous iron components during the co‐precipitation of magnetite

Previous reports support the finding that mixed iron oxide populations are often obtained for RTCP. This is seen for example in the control sample TEM images presented in Amemiya et al.17a However, in the presence of Mms6, Amemiya et al. showed that the homogeneity of the sample was greatly enhanced with respect to both the iron oxide phase (controlled for magnetite, with removal of undesired iron oxide by‐products) and narrowing of the size distribution of magnetite nanoparticles. It therefore appears that Mms6 can control the formation of the specific mineral magnetite, as well as its size.

**Figure 3:**
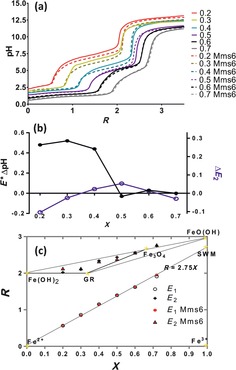
a) pH titration profiles for the addition of Mms6 at various values of *X*. b) Graph to show how the Mms6 and control pH titrations vary at different *X* values. Left axis and black closed circles show relative pH difference at E* (central plateaus), right axis and open blue circles shows difference in *E*_2_. Data show the greatest difference at lower values of *X* (ferrous‐rich solutions). c) Mass‐balance obtained from titration data with Mms6. Red circles show the *E*_1_ values for reactions with Mms6: the position is close to the experiments without protein. The red triangles show the *E*_2_ values in the presence of protein. Black open circles are *E*_1_ without protein and crosses are *E*_2_. The errors are too small to be shown (*E*_1_=zero (to 3 decimal points) and *E*_2_=±1.3 %). The positions of various iron oxide minerals are indicated in the diagram. GR=green rust and SWM=schwertmannite.

To study Mms6 further we added purified protein to the reaction at a concentration of 10 μg mL^−1^ of reaction solution. The pH titration profile was recorded at various values of *X*, shown in Figure 3 a. The *R* positions of equivalence points *E*_1_ and *E*_2_ were plotted on the mass‐balance diagram to allow comparison between the control titration and the corresponding Mms6 experiment (Figure 3 c).3

From these pH data we can see that there is negligible difference between the titrations at low pH, and thus the *R* values of *E*_1_ do not alter between the control and Mms6 experiments, with all falling very close to this ideal line for the formation of schwertmannite, in agreement with previous control studies.19a

Results in the literature have shown that Mms6 is able to bind ferric ions.^7^, ^8^ However, this is not noticeably the case in this study and as such we propose that Mms6 may only bind to ferric ions at higher pH, or if there is no competing ferrous ions, or that the binding to soluble ferric ions is at concentrations too low to detect in this titration. It should be noted that the methodology used in this study is likely to only detect the formation and precipitation of solids from solution and is unlikely to detect the binding of iron ions by Mms6 alone. The consistency between control and Mms6‐mediated RTCP reactions at *E*_1_ indicates Mms6 does not alter the balance of iron species present at low pH.

With Mms6 present, the formation of the second plateau occurs earlier and at lower pH for *X*≤0.4 (Figure 3 a and b). This shows that Mms6‐containing reactions begin to form new precipitates at a lower pH after the same quantity of base has been added compared to the control reactions. This indicates that in the presence of Mms6, Fe^2+^ and hydroxide ions combine more readily with the ferric oxyhydroxide to generate the mixed valence iron minerals magnetite and green rust.^19^ Here it should be noted that magnetite precipitates at a lower pH than green rust [see Eqs. (2) and (3)], thus comparably more magnetite is being precipitated at this second plateau in the Mms6 mediated reactions than the controls for *X*≤0.4, demonstrating that Mms6 is able to direct the nucleation and formation of magnetite preferentially. The difference becomes negligible when the reaction is performed under conditions where the ferric and ferrous iron are equally balanced or ferric ions predominate (Figure 3 a and b).

**Figure 4:**
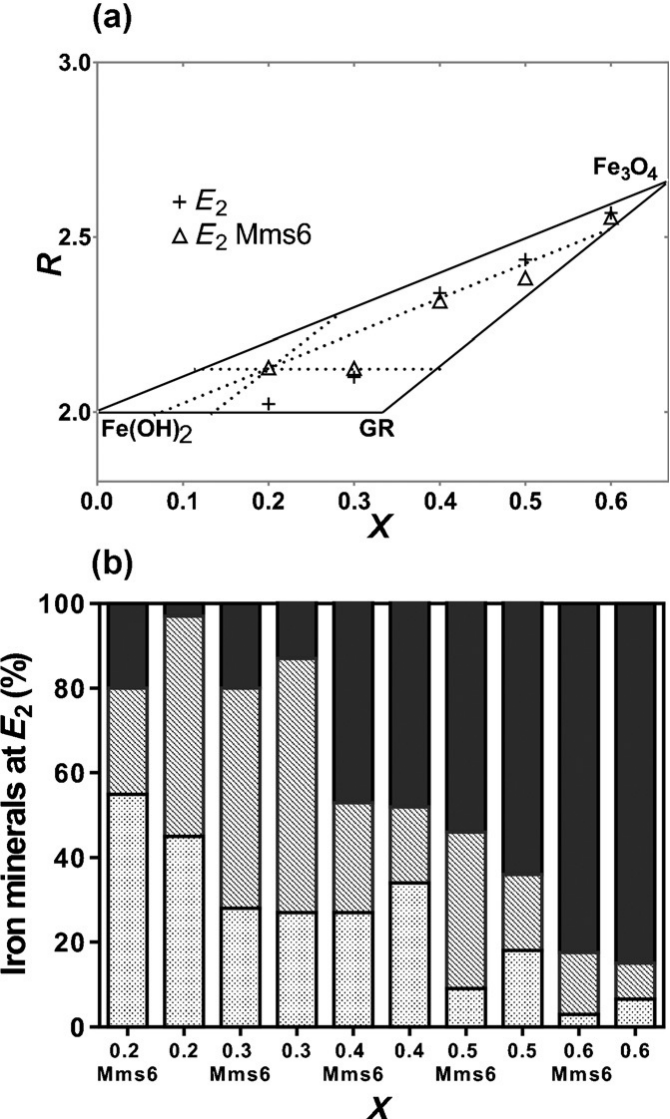
a) Detailed view of the upper part of the mass balance diagram. Triangles are *E*_2_ values of the Mms6 reactions and black crosses are protein‐free *E*_2_ values. As an example, black dotted lines represent bisections of the phase diagram to calculate the quantities of each iron mineral for the *X*=0.3 Mms6 data point. b) The percentage of each iron mineral calculated from the mass balance diagram is shown for each value of *X* either with or without Mms6. Dotted boxes=ferrous hydroxide, hashed boxes=green rust species, and black boxes=magnetite.

For each value of *X*, from the *E*_2_ position in the mass‐balance diagram (Figure 3 c, Supporting Information Table S1) the relative amounts of each of the possible minerals (ferrous hydroxide, green rust and magnetite) were calculated (Figure 4 a; note 0.2≤*X*≤0.6 as the higher *X* point is outside this mass balance mineral regime and shows negligible difference between control and Mms6 samples). These relative amounts are shown in Figure 4 b (values in Supporting Information Table S2). At *X*=0.2 (the most extreme ferrous‐rich ratio tested) the control reaction products are dominated by green rust and ferrous hydroxide species (as the excess ferrous ions are precipitated) with negligibly small quantities of magnetite being produced. However, at this same *X* value with the addition of Mms6, approximately 20 % of the mineral species formed is magnetite, a similar quantity to the green rust, consuming the available ferric ions. Our data show that Mms6 is acting to promote the formation of magnetite at lower *X* values. This magnetite increase is larger in the ferrous iron rich reactions (i.e., when *X* is lower), with the quantity of magnetite coinciding with the protein free reactions at around *X*=0.4. Then at higher values of *X* there is less effect on the quantity of magnetite formed (as this is able to occur more readily anyway). However, more green rust is formed at the expense of ferrous hydroxide (compared to the control), and this is more able to mature and convert to magnetite under these conditions (Figure 4 b). Furthermore, the magnetic data follow this trend (Supporting Information Figure S3 and Table S4

Taken together, our analysis indicates that the main role of Mms6 is to sequester and bind ferrous ions, particularly at the later, higher (post *E*_1_) pH stages where they would normally start to precipitate. The result of this activity is that Mms6 promotes the formation of magnetite under unfavourable ferrous‐rich conditions when it is less able to yield magnetite as a product in the control reaction (Figure 4 b). Mms6 could thus be considered as acting as a “mineral/ferrous ion buffer” seemingly enhancing the propensity to nucleate magnetite, even when this does not normally occur, as in the case where *X*=0.2, and directing its precipitation towards magnetite rather than other mixed valence or ferrous salts.

### Ferrous and ferric iron interactions with Mms6‐derived peptides

Despite extensive study there is currently no structural information on Mms6 or its C‐terminal iron‐binding domain. We have attempted protein crystallization and NMR spectroscopy on the full‐length protein but this has proved intractable due to protein aggregation and disordered structures. However, in order to obtain structural information relating to metal binding, the whole protein (particularly the proposed hydrophobic self‐assembly part) is most likely not required. The C‐terminal region is the most acidic and thus proposed to be the iron binding site. There is also some evidence that the C‐terminal region alone can show some activity in magnetite formation reactions, suggesting it probably retains the ability to bind iron ions even when the remaining two thirds of the full length protein is removed.^12^ In order to address the questions of the metal‐binding properties of the protein we have employed a 2D NMR spectroscopic approach to investigate possible structure and iron binding characteristics (particularly and uniquely considering ferrous iron binding) of a 20 amino acid peptide sequence consisting of the acidic region of Mms6 under physiological pH conditions. We designate the Ac‐KSRDIESAQSDEEVELRDAL‐Am peptide as C20Mms6 (N and C terminus were modified by acetylation and amidation, respectively, to negate possible interactions with the termini which would not appear in the full‐length protein at these residue positions). Crucially the C‐terminal region does not have the same micellar‐forming properties as full‐length Mms6, which simplifies its study by NMR spectroscopy.

**Figure 5:**
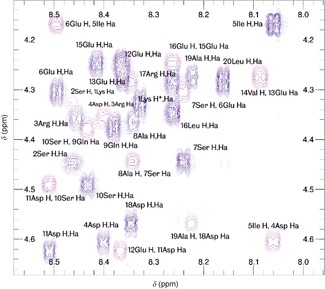
A portion of the HN‐Hα region for C20Mms6 from TOCSY (blue) and ROESY (purple) experiments at pH 7.

We performed TOCSY and ROESY NMR experiments on the C20Mms6 peptide in sodium phosphate buffer at pH 7 (pH of the species formed at the second plateau where we see Mms6 activity) in order to obtain a complete assignment of the peptide for the backbone plus sidechains as far as Hβ or Hγ. An assignment can be found in Supporting Information Table S4 and a portion of the spectrum can be seen in Figure 5. The 2D NMR studies of the C20Mms6 peptide sequence do not show any evidence for a defined structural conformation in the absence of iron. We did not observe any significant chemical shift differences from random coil values^22^ (largest Hα difference from expected random coil values is 0.092 ppm, mean Hα difference is 0.021(±0.028) ppm; complete details can be found in Supporting Information Figure S4). This indicates that the peptide adopts an unstructured conformation in solution. In addition we did not observe any non‐sequential NOE peaks due to through‐space interactions between side chains.5

The presence of iron ions in NMR experiments severely suppresses peak intensities through paramagnetic relaxation enhancement (PRE). This mechanism is a result of interaction of the unpaired electrons of iron with the NMR active nuclei. PRE can be a useful tool in structural biology for identifying residues of a metalloprotein which are involved in binding paramagnetic metals,^23^ but is undesirable here because it leads to signals becoming broad and unobservable. Hence we used low peptide concentrations which allow the addition of extremely small quantities of iron to keep PREs to a minimum. This strikes a good balance between maintenance of the signal level and longer acquisition times. Furthermore, addition of iron to peptide samples acts to lower the pH which can show a shift. These can mask the chemical shift changes due to metal binding. We therefore included in excess a non‐chelating buffer, MES, to strictly regulate the pH during the experiment and we checked the pH carefully before and after addition of metals and adjusted it where necessary, to ensure that any differences we observed were in fact due to metal binding rather than pH changes.

**Figure 6:**
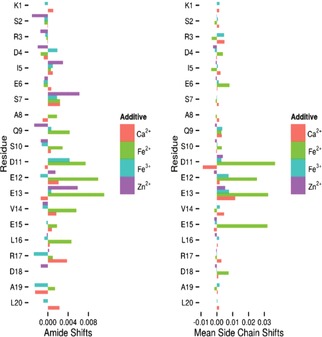
Chemical shift changes for C20Mms6 peptide with the addition of 3 equivalents of metals to 50 μm peptide, Fe^2+^, Fe^3+^, Ca^2+^, Zn^2+^. Left: amide protons; right: mean sidechain shifts.

Previously reported studies have shown that Mms6 can bind Fe^3+^ as well as other metals such as calcium and magnesium, but not copper, manganese or zinc.^7^ Based on this evidence we screened a panel of metals consisting of Fe^2+^, Fe^3+^, Ca^2+^, and Zn^2+^. This allowed us to compare metals which C20Mms6 either should bind (Fe^3+^, Ca^2+^) or should not bind (Zn^2+^) and to test the binding of the unknown Fe^2+^. Considering that magnetite is composed of an ordered mixture of both ferrous and ferric ions, and from the insight we have obtained from the pH titrations, this experiment was designed particularly to probe Fe^2+^ interaction and binding as there has been no previous analysis of Mms6 binding to ferrous iron. Ferrous and ferric iron chloride were tested in separate NMR experiments by titrating iron in small increments into the NMR tube until the signal levels were too small to be detected, indicating the maximum quantity that could be added. To interpret the C20Mms6 chemical shifts upon addition of metal ions we analysed both the shift of the amide proton peaks, Figure 6 a, as well as the mean shift value for the side chain signals, Figure 6 b. As we expected, the addition of zinc to the C20Mms6

Addition of either ferric or calcium ions, both predicted to bind, did result in a number of significant chemical shift changes. The most shifted residues are located at the acid‐rich cluster D11–E13 with the largest average sidechain shift recorded for ferric binding at the sidechain of glutamic acid E13. However, the changes were small. Furthermore, losses in signal intensity due to PREs from Fe^3+^ were widely distributed along the peptide sequence, further implying nonspecific binding of Fe^3+^.

The most significant shifts and intensity changes were observed upon the addition of ferrous iron. The magnitudes of the shifts in this case are up to fivefold greater than those observed for ferric addition. Residues D11, E12, E13, and E15 are all implicated in binding as well as (more weakly) E6 located further away on the polypeptide chain. This clearly indicates that C20Mms6 is able to specifically coordinate Fe^2+^, through the carboxylate side chains of the five key residues mentioned above, though presumably not all at the same time. The amide groups of the peptide main chain (Figure 6 a) also show significant shifts, potentially indicating that the backbone amide of these residues may be involved in binding the metal ions directly or that the chemical environments of the amide backbones have changed as a result of binding through the side chain groups. The latter reason seems more likely as it implies some conformational change of the peptide as it coordinates ferrous iron with several sidechains simultaneously.

### Simulation studies

**Figure 7:**
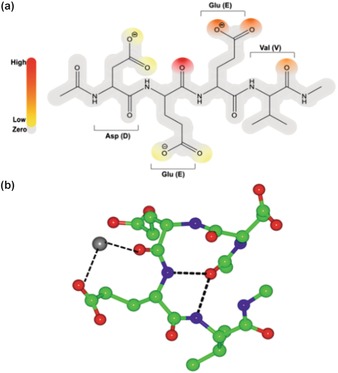
Simulated ferrous iron binding to acid‐rich DEEV cluster of Mms6. a) Locations and probability of binding of ferrous iron: red is high probability, orange medium, and yellow low. Grey represents areas of no binding. b) Typical snapshot of peptide binding to ferrous iron (grey sphere). Black dashed lines are hydrogen bonds, in this example between Fe^2+^ and both E12 carbonyl and E13 sidechain, and hydrogen bonds from the N‐cap carbonyl to both E13 NH and V14 NH.

To complement the experimental study, and gain further insight at the molecular level, a model peptide (DEEV) was subjected to molecular dynamics (MD) simulations using DL_POLY classic^24^ to investigate ferrous iron binding. DEEV is the region of Mms6 that displays significant metal binding in our NMR experiments. Interactions were explored with 2 ns MD simulations between DEEV and a single ferrous ion placed initially at one of 12 potential binding sites (Supporting Information Figure S5). The position of the ferrous ion during each simulation was tracked relative to the oxygen atoms of the peptide to determine the strength of the interaction and the most likely binding sites. We found that 16 % of the sampled configurations resulted in ferrous binding, defined as the metal ion being positioned within 3 Å of at least one oxygen atom of the peptide. A more detailed picture of binding was obtained by considering which of the oxygen atoms within the peptide were most likely to be involved in ferrous binding. These results are summarised in Figure 7 a.7

We find that one of the most favoured sites is the carboxylic acid sidechain of the second glutamic acid (E13). However, the most significant binding from the simulations is with the main chain carbonyl, located between the two glutamate (E12–13) residues, with 56 % of the simulations that resulted in ferrous binding displaying interactions with this carbonyl. Similarly, the NMR data report large chemical shifts of the amide protons for both of these flanking glutamate residues, which could be due to Fe^II^ interaction with the peptide backbone of this region. However, it could also be due to multiple bonds between the oxygen atoms in the peptide and the ferrous ion. Further analysis of the MD trajectories when the ferrous ion is bound to the backbone in the (E12–13) region reveals that in 90 % of these occurrences it is also bound to one or other of the oxygen atoms in E13 carboxyl group, compared to just 6 % of the configurations where it is bound to the oxygen in the backbone only. We do not observe significant binding to the aspartate residue (D11) in our simulations. However, in our simulation, the peptide adopts a more compact arrangement upon ferrous binding, with hydrogen bonds present between the amide protons of the glutamate residues and the amide carbonyl of the N‐terminal capping group (Figure 7 b). This rearrangement is likely due to the short length of the peptide, but could indicate that, whilst not participating in ferrous iron binding directly, these residues may undergo significant movement upon addition of metal ions, leading to the chemical shift changes seen.

We also observed that when the ferrous ion is replaced with ferric ion in the simulations, the additional positive charge makes binding to the peptide more favourable, but also means iron binding is seen indiscriminately at all the oxygen sites present. We believe it is likely that the additional selectivity shown by ferrous ions may explain why larger and more specific chemical shift changes are detected experimentally in the ferrous system.

**Figure 8:**
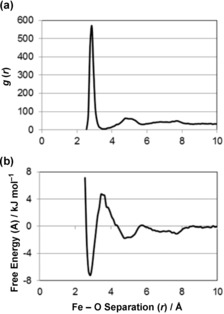
a) The radial distribution function (RDF) for the ferrous ion interacting with all oxygen atoms in the peptide, and b) transformation as a free‐energy profile of iron binding as a function of distance from oxygen sites at 300 K.

The ferrous–oxygen radial distribution function (RDF; Figure 8 a) effectively provides a summary of the time spent by the ferrous ion at a particular distance from an oxygen atom within the peptide and gives us an indication of the stability and strength of binding (Figure 8 b).8

The RDF represents the combination of a number of simulations, each searching a different part of configurational space. Therefore, it is reasonable to interpret the intensity of the RDF as the probability of a ferrous ion being at a particular distance from an oxygen atom, making it a good approximation for the partition function of the system. The RDF can be transformed into a plot of free energy versus iron–oxygen separation using the statistical thermodynamic relationship:

A=-RTlnQ=-RTln(g(r))

where *g*(*r*) is the radial distribution function (Figure 8 a). The RDF will converge to a value of unity at large separations, meaning the calculated free energy will be zero when the separation is large and that the calculated values represent the free energy change in bringing the ferrous ion towards the peptide to a separation, *r* (Figure 8 b).

Inspection of Figure 8 a shows that the binding to the peptide occurs over a narrow range of separations between 2.55 and 3.25 Å (optimum bond distance 2.85 Å). The intensity of this peak, and the narrow range of bond distances it covers, suggests that when iron is bound it is held relatively strongly. The fact that the RDF drops close to zero between the first and second peak illustrates that once a ferrous ion binds to a particular site, it will remain bound to the peptide for a significant time in most cases.

These observations are clearer when the data are plotted as an energy profile (Figure 8 b). A deep energy well is evident close to the surface which corresponds to a binding energy of −6.7 kJ mol^−1^ (−2.7 *RT*). This is sufficiently large that it could not be overcome by the kinetic energy possessed by the atoms. Therefore, once a ferrous iron has entered the well it is unlikely to leave during the simulation. To leave the adsorption layer, the ion must also cross the energy barrier at 3.5 Å separation, giving a total barrier to desorption of 10.2 kJ mol^−1^ (4.0 *RT*).

Whilst indicative of the nature of binding, it must be noted that Figure 8 is an average of the behaviour of a ferrous ion in multiple simulations and does not give any indication of how the energy profile differs at sites where iron binding is known to be favoured and where it is not observed.

Such insight can be gained by plotting the RDF (and associated free‐energy profile) for the individual interactions between a particular oxygen and the ferrous ion (Supporting Information Figures S7 and S8). At residue E13 and the bridging carbonyl oxygen between E12 and E13 sites, the binding energy is significantly higher, between −9 and −11 kJ mol^−1^ (or 3.5 to 4.5 *RT*), with the additional energy required for the ferrous ion to leave the adsorption layer approximately 4 kJ mol^−1^ (≈14 kJ mol^−1^). This suggests that once an ion enters these binding sites it will not leave for the duration of the MD simulation and that binding should be observable on an experimental timescale. The sites in D11 and E12, where limited iron binding is seen in the MD simulations, display a binding energy of approximately −4 kJ mol^−1^_._ This is close to the kinetic energy possessed by the atoms in ambient conditions. An additional energy barrier must still be crossed for the ion to completely leave the binding site but is still of an order that such desorption is possible and hence why only limited binding is observed in the MD simulations. When extended to larger time scales, this would represent a system where continuous exchange between binding and desorption would be possible, especially when concentration effects, not present in our MD simulations, are taken into account.

Similar calculations were performed using Fe^3+^ instead of Fe^2+^. The additional charge on the iron made the overall binding energy to this negatively charged peptide more favourable. However, it is significant that binding was less specific to E12/E13.

## Discussion and Conclusions

Analysing the effect of Mms6 in situ through pH measurements throughout the magnetite‐formation process has enabled us to obtain an insight into the protein's function and in particular, at what stage in the reaction it exerts its effect. We see no influence of the protein in the early stages of the reaction at low pH at the point where ferric oxyhydroxide minerals are formed, consistent with the theoretical isoelectric point of 4.2 of the acidic iron binding region of the protein. It is from pH 5 and upwards that we see Mms6 affecting the formation of the minerals, at the point that ferrous ions begin to precipitate out of solution and mixed valence iron minerals are formed. From our data it appears that Mms6 promotes the formation of magnetite under ferrous‐rich conditions, even in conditions in which magnetite does not readily form. All previous studies on the iron binding activity of Mms6 have focused on ferric‐ion binding of the acidic C‐terminal region^7^, ^8^ and often at low pH. From our study it seems Mms6 is more likely to interact predominantly with ferrous ions at pH>5. In this context we performed an NMR metal‐binding experiment under these conditions and we saw that Mms6 does indeed show specific and significant interactions with ferrous ions compared to both positive and negative control metals, including ferric ions. The residues responsible for binding metals vary for ferrous and ferric ions, but the DEEVE cluster was found to have the biggest shift for both iron ions, with the double EE site having the prominent shift for ferric ions and all the acidic residues of the whole DEEVE region significantly shifted (up to 5×>Fe^3+^) in the presence of ferrous ions.

The interaction of this smaller peptide of interest with ferrous and ferric ions was thus modelled by atomistic simulation in order to further understand the binding event. This indicated that the acidic residues may bind the ferrous iron not only through their carboxylate sidechain but also through the peptide carbonyls. The MD calculations also show that multidentate binding is preferred, especially for ferrous ions, and that bidentate binding of Fe^2+^ to E12 carbonyl and E13 carboxylate is particularly frequent. Ferric ions show binding, but unspecifically across the acidic amino acids.

As the pH is raised, and particularly at high ratios of ferrous to ferric ions, the initially formed ferric oxyhydroxide precipitate must re‐dissolve and reform into a different crystalline structure in order to incorporate ferrous ions and form magnetite. Our data suggest that Mms6 is facilitating this process. We therefore propose an in vitro mechanism in which Mms6 displays little activity below pH 4–5. Above this, Mms6 specifically sequesters and binds ferrous ions. We also propose that the Mms6’s known affinity for ferric ions promotes the dissolution of the unstable ferric precursors to sequester ferric ions as well. We have shown here that the role of Mms6 is to promote the formation of magnetite at high ferrous ratios, which are conditions where magnetite is formed poorly if at all in the absence of Mms6. We therefore propose that Mms6 not only helps to dissolve undesired precipitates, such as ferric oxyhydroxide, but helps the formation of magnetite by acting as a nucleation protein.

Mms6 is known to self‐aggregate^10^ and it is already proposed that Mms6 assembles to form an acidic, charged, C‐terminal surface to bind multiple iron ions.^8^, ^25^ The self‐assembly of Mms6 could space these acidic residues to bind both ferrous and ferric ions in a magnetite crystallographic geometry to template and promote the initial formation of magnetite over other iron minerals by lowering the free energy for formation of magnetite.

Relating the reactions occurring in solution in this experiment to those found in vivo is challenging. Mms6 is associated with the magnetosome membrane, so the C‐terminal acidic surface of an Mms6 assembly will differ in its in vitro micelle structure compared to the arrangement in the internal lumen of the magnetosome. In the magnetosome there are transporters that transport iron ions into the magnetosome.^26^ All current knowledge points to the transport of ferrous ions only, with partial oxidation of some of the ferrous ions to generate the ratio required for magnetite formation.^26^ Furthermore, ferrous transporters are antiporters meaning they bring ferrous iron in as they take protons out. This will help to regulate the internal pH, but the precise pH of this environment is also not currently known. Additionally, due to the iron transport system it is highly unlikely that there will be SO_4_^2−^ counter‐ions, ruling out the formation of schwertmannite and green‐rust phases.

However, similarities between the two systems can be drawn to predict the action of Mms6 in the magnetosome. While the pH of the interior of the magnetosome has not yet been determined, it must at some point be high enough to enable magnetite to crystallise (≥pH 7), and we have established in this study that Mms6 is active in these conditions. In the natural system we assume some of the ferrous ions are converted to ferric (through the action of oxidase enzymes), and thus a ferric mineral will precipitate (due to its chemical insolubility at pH≥2.5). There have been several reports of such ferric precursors,^27^ and while our suggested mechanism relies on dissolution of this precursor, its exact form is immaterial, be it schwertmannite (in vitro) or ferrihydrite (in vivo). Interestingly, Mms6 activity is greatest in ferrous‐rich conditions, which is likely to be the predominant state in magnetosomes if it is confirmed that iron transport is of ferrous iron. It is therefore likely that in vivo conditions in the magnetosome are similar to our in vitro conditions with respect to possible pH, ferric precursors in a ferrous‐ion‐rich solution, and the protein's ability to form a self‐assembled charged surface capable of sequestering ferrous and ferric iron in the exact proportions to promote the crystallization of magnetite.

Therefore we propose, both in vitro and in vivo, that Mms6 is a magnetite nucleation protein, able to bind both ferric and specifically ferrous iron to initiate the process of templating the magnetite crystal.

## Experimental Section

Ferrous and ferric sulfate were purchased from Sigma–Aldrich and used without further purification. ICP‐AES was used to check the hydration level of both compounds prior to each set of experiments.

Peptide C20Mms6 KSRDIESAQSDEEVELRDAL, featuring an acetylated N terminus and an amidated C terminus, was purchased from Genscript (USA). The peptide was dissolved in and dialysed, overnight, against a chosen buffer in a dialysis cassette with a MWCO of 1 kDa, to remove trace salts. MES and NaH_3_PO_4_ were purchased from Sigma–Aldrich and used without further purification.

### Biochemical methods

The *mms6* sequence from *Magnetospirillum magneticum* AMB‐1 was introduced into a pTTQ18‐based expression vector by cohesive‐end cloning with the resulting plasmid, pHis8mms6, encoding N‐terminally octahistidine tagged Mms6. The protein was produced in *E. coli* BL21 star (DE3) cells (Invitrogen) harbouring a pRARE (Merck) plasmid to compensate for codon bias in the *mms6* sequence. Cells were cultured in autoinducing Superbroth (Formedium) at 37 °C with shaking for 24 h in the presence of carbenicillin and chloramphenicol to select for the pHis8mms6 and pRARE plasmids, respectively. Cells were lysed by sonication in 25 mm Tris pH 7.4, 100 mm NaCl. The insoluble material, containing the His8–Mms6 inclusion bodies, was collected by centrifugation at 16 000 *g* and resuspended in 6 m guanidine hydrochloride, 25 mm Tris pH 7.4 to solubilise the proteins. Further centrifugation at 16 000 *g* was performed to remove any material not solubilised by the guanidine treatment. The supernatant was mixed with nickel charged nitrilotriacetic acid (NTA) resin (Amintra resin, Expedeon) to allow binding of the histidine tagged Mms6. The resin was subsequently packed into a gravity flow column and washed extensively with wash buffer (6 m guanidine hydrochloride, 25 mm Tris pH 7.4, 10 mm imidazole) before elution of the bound protein in 300 mm imidazole‐supplemented wash buffer. The eluted protein was refolded by rapidly diluting into a large volume of refolding buffer (500 mm NaCl, 25 mm tris pH 7.4) before being concentrated using a 10 kDa molecular weight cut‐off centrifugal concentrator (Sartorius). The concentrated material was subjected to centrifugation to remove any small amounts of precipitated protein before dialysis against 500 mm NaCl using a 3.5 kDa molecular weight cut‐off slide‐a‐lyser (Thermo Scientific). The refolded His8–Mms6 was quantified by absorbance at 280 nm and stored at 193 K.

### NMR spectroscopy experiments and analysis

For NMR studies all samples were prepared in a 90 % H_2_O/10 % D_2_O solvent mixture. NMR spectroscopy was performed on a Bruker Avance DRX instrument at 600 MHz, equipped with a triple resonance (^1^H‐^13^C/^15^N‐^2^H) cryoprobe with *z*‐gradient, at 25 °C. TOCSY experiments were carried out with a spin lock power of 8.3 kHz for 45 ms, and ROESY experiments with a spin‐lock power of 2.27 kHz with a 200 ms mixing time. Solvent suppression was achieved with a 3–9–19 pulse sequence with presaturation during the relaxation delay of 1.5 s for the TOCSY experiment, and with excitation sculpting with gradients for the ROESY experiment.

For sequential assignment, 1 mm C20Mms6 was prepared in 20 mm NaH_3_PO_4_ buffer at pH 7. 3‐(Trimethylsilyl)‐2,2′,3,3′‐[D_4_]‐propionate (TSP) was added as a standard (0.0 ppm). For metal binding studies, 50 μm C20Mms6 was dialysed extensively against MES buffer (50 mm) at pH 5.66±0.02. Metals were dissolved in the same buffer, and added directly to the NMR tube stepwise until the metal concentration was 150 μm. At each addition, the pH was checked and adjusted to 5.66±0.02 if necessary.

### Co‐precipitation of magnetite

Ultrapure (MilliQ) water was degassed under vacuum and sparged with nitrogen for at least 1 h to remove dissolved oxygen. An aliquot (20 mL) of this water was added to a three‐necked glass flask. The solution is isolated from the air by continuous sparging with nitrogen. This is also used to mix the solution. Fe salts were measured to 0.1 mg accuracy and stored in a small plastic tube. Initially Fe^3+^ was added to the solution by removing 1 mL of the solution and dissolving the Fe salt in the tube. This was then added to the solution with a glass pipette. Fe^2+^ was then added in the same way. Each set of experiments varies the *X* parameter from 0.2 to 0.5. One batch of nitrogen sparged 1 m NaOH solution was used per set of experiments to ensure consistency.

A Mettler Toledo 7 Multi‐pH meter with Micro‐Pro probe was immersed in the solution. Data were logged on a computer via serial port communication at three second intervals for the duration of the experiment. The NaOH injection needle was pre‐primed before the start of the experiment. This solution was added continuously to the reaction flask at a rate of 50 μL min^−1^ via a syringe pump driver.

### Simulation studies

The molecular dynamics simulations were based on a molecular mechanics approach where the intra‐ and intermolecular interactions within the peptide were described using the generalised Amber force field (GAFF)^28^ which also incorporates a version of the TIP3P water molecule.^29^ Partial charges on the peptide were determined using semi‐empirical calculations at the AM1 level of theory.^30^ A total of 24 production calculations were performed (2 different ions with 12 starting configurations) where the peptide was placed at the centre of a 40 Å×40 Å×40 Å box which contained approximately 2000 water molecules. In all cases the 3D boundary conditions were applied within the isobaric isothermal NPT ensemble which allows the sizes of the simulation cell to change during simulation. The temperature and pressure were maintained at 300 K and 10^5^ Pa using a Nose–Hoover thermostat and barostat with a period of 0.1 and 0.5 ps.^31^ The trajectories were generated using the Verlet leapfrog algorithm with a time step of 1 fs.^32^

## Supporting information

As a service to our authors and readers, this journal provides supporting information supplied by the authors. Such materials are peer reviewed and may be re‐organized for online delivery, but are not copy‐edited or typeset. Technical support issues arising from supporting information (other than missing files) should be addressed to the authors.

Supplementary
