# Supplementary material for: Ferrous Iron Binding Key to Mms6 Magnetite Biomineralisation: A Mechanistic Study to Understand Magnetite Formation Using pH Titration and NMR Spectroscopy
Source: Chemistry. 2016 Apr 26;22(23):7885–94. doi: 10.1002/chem.201600322 (PMC5082532; doi:10.1002/chem.201600322)
Supplement: Supplementary file 1 — Supplementary [file CHEM-22-7885-s001.pdf]

CHEMISTRY
A European Journal

Supporting Information

Ferrous Iron Binding Key to Mms6 Magnetite Biomineralisation: A Mechanistic Study to Understand Magnetite Formation Using pH Titration and NMR Spectroscopy

Andrea E. Rawlings,[a] Jonathan P. Bramble,[a] Andrea M. Hounslow,[b] Michael P. Williamson,[b] Amy E. Monnington,[c] David J. Cooke,[c] and Sarah S. Staniland*[a]

chem_201600322_sm_miscellaneous_information.pdf

Ferrous iron binding key to Mms6 magnetite biomineralisation: A mechanistic study to understand magnetite formation using pH titration and NMR.

Andrea E. Rawlings,[a] Jonathan P. Bramble,[a] Andrea M. Hounslow,[b] Michael P. Williamson,[b] Amy E. Monnington,[c] David J. Cooke,[c] and Sarah S. Staniland*[a]

Supplementary figures

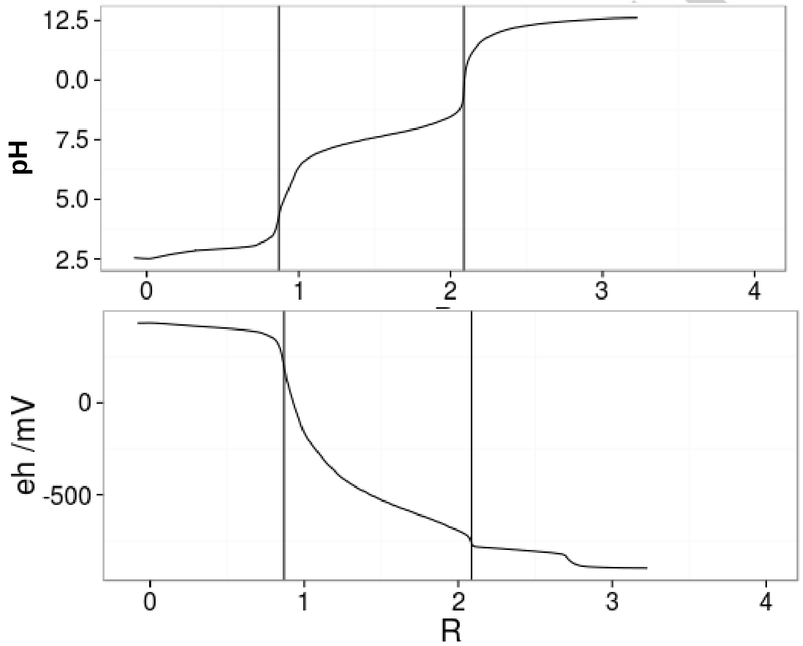

Figure S1. pH and Redox potential of the titrations as a function of R for X= 0.3.

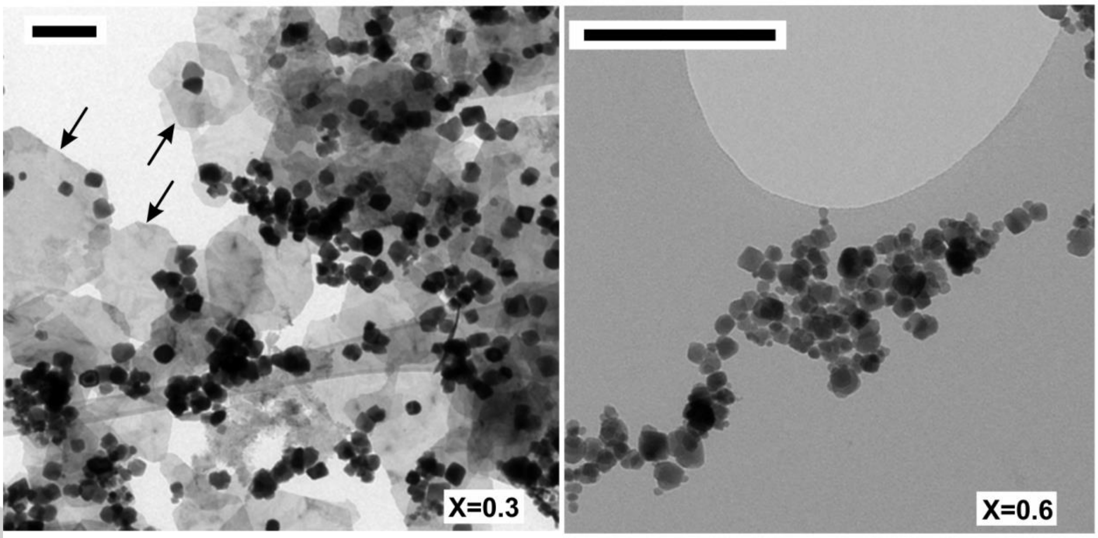

Figure S2. TEM images of particles at X=0.3 and X=0.6. Hexagonal plate structures identified as green rusts are indicated in the X=0.3 image with arrows. Scale bars are 250 nm in each image. Note nanoparticles are much smaller for X=0.6. This clearly shows that the ratio of iron salts is critical for control over nanoparticle size and composition.

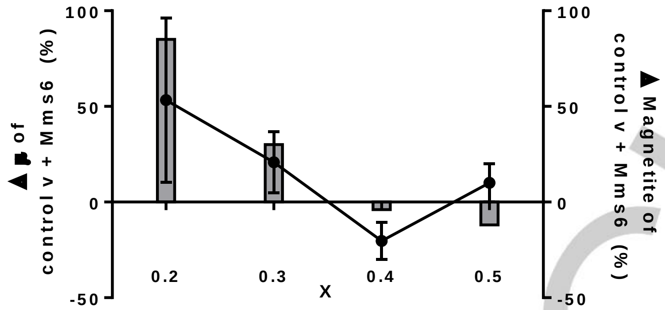

Figure S3. The magnetic moment was measured for mini precipitation samples at R = 1.76 (coinside with the second plateau of NaOH for value of X (0.2-0.5) for both control precipitations and those supplemented with Mms6; to correlate with the second plateau (Figure 3a) and the mineral proportions depicted in Figure 4b and table S2. Due to the very small values (especially at low X) the data is shown as the difference between Mms6 supplemented samples and control samples as a % of the value (-•-). For comparison the difference between the quantity of magnetite (obtained from Figure 4b and table S2) between the Mms6 supplemented samples and control samples at each value of X is represented in the same way (grey bars). (Values are given in table S3).

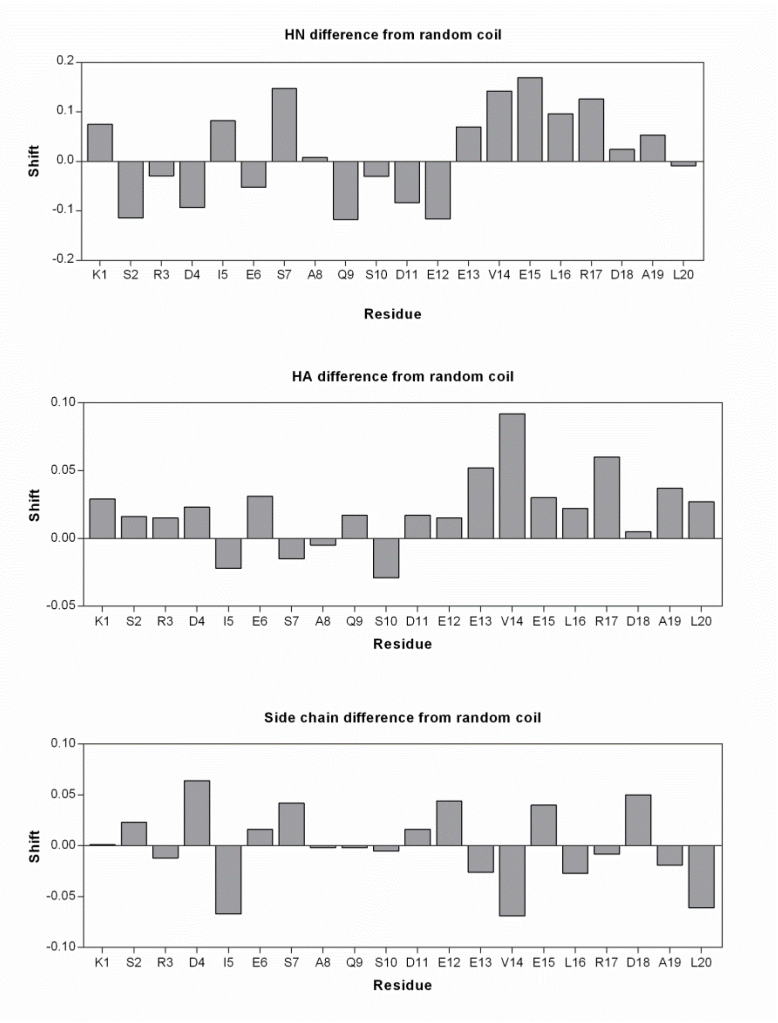

Figure S4. Differences from random coil positions for C20Mms6. Table S3 contains the source data.

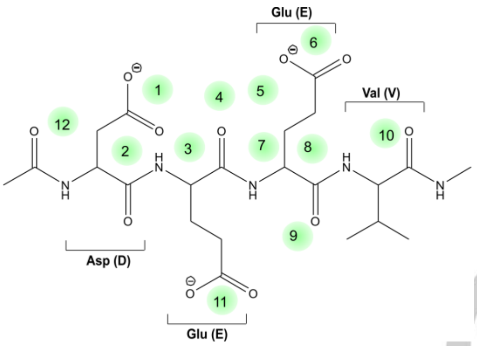

Figure S5. Position of the initial ferrous ions (green) in relation to the DEEV peptide used in the moelcular dynamics simulations (numbered 1-12).

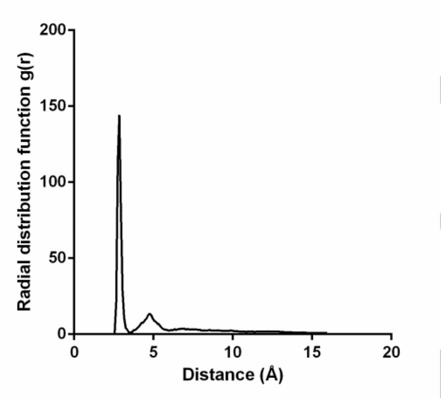

Figure S6. Mean radial distribution function for oxygens binding to the ferrous ion in Figure 7b.

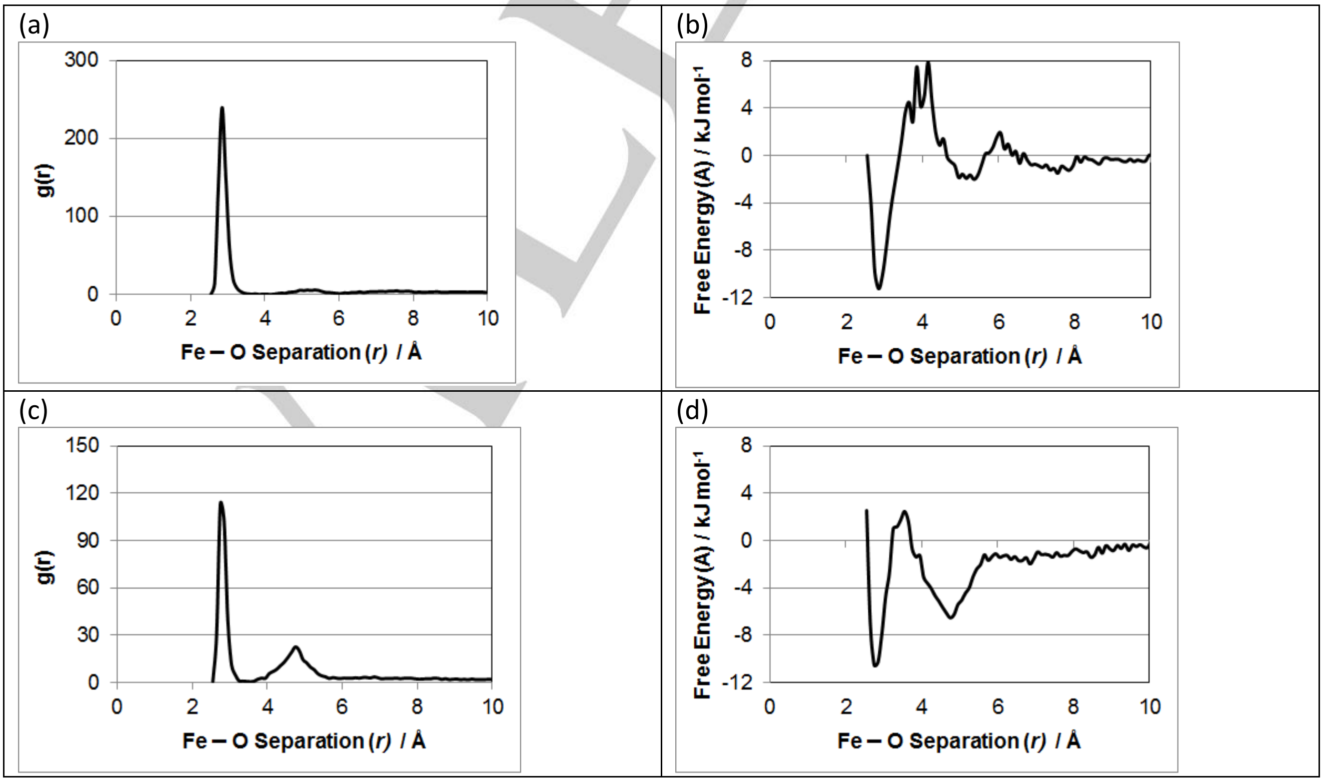

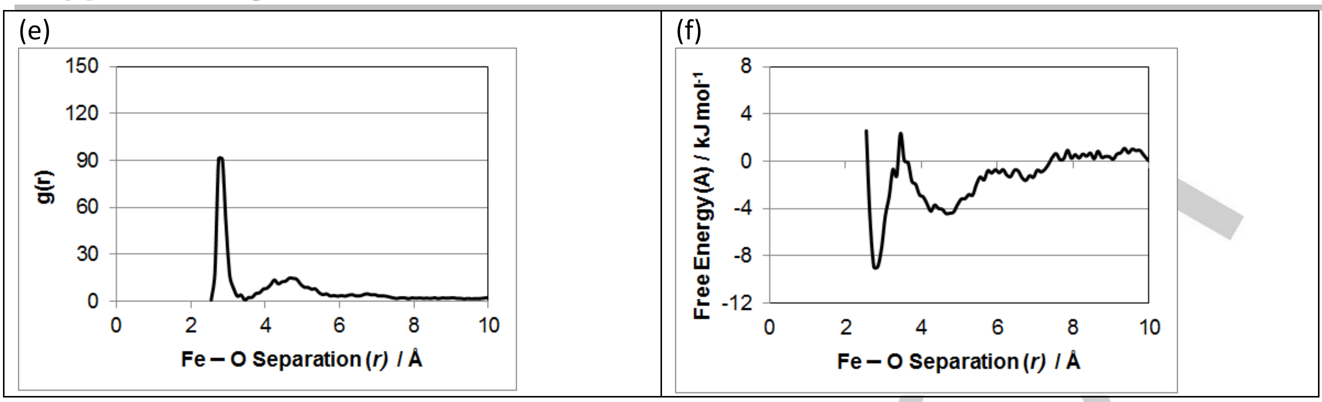

Figure S7: Fe--O radial distribution functions and free energy profiles at 300 K for the main sites where binding is observed in the MD simulation: a) and b) the backbone oxygen between E12 and E13, c) –f) the oxygen atoms in the GLU group at E13. .

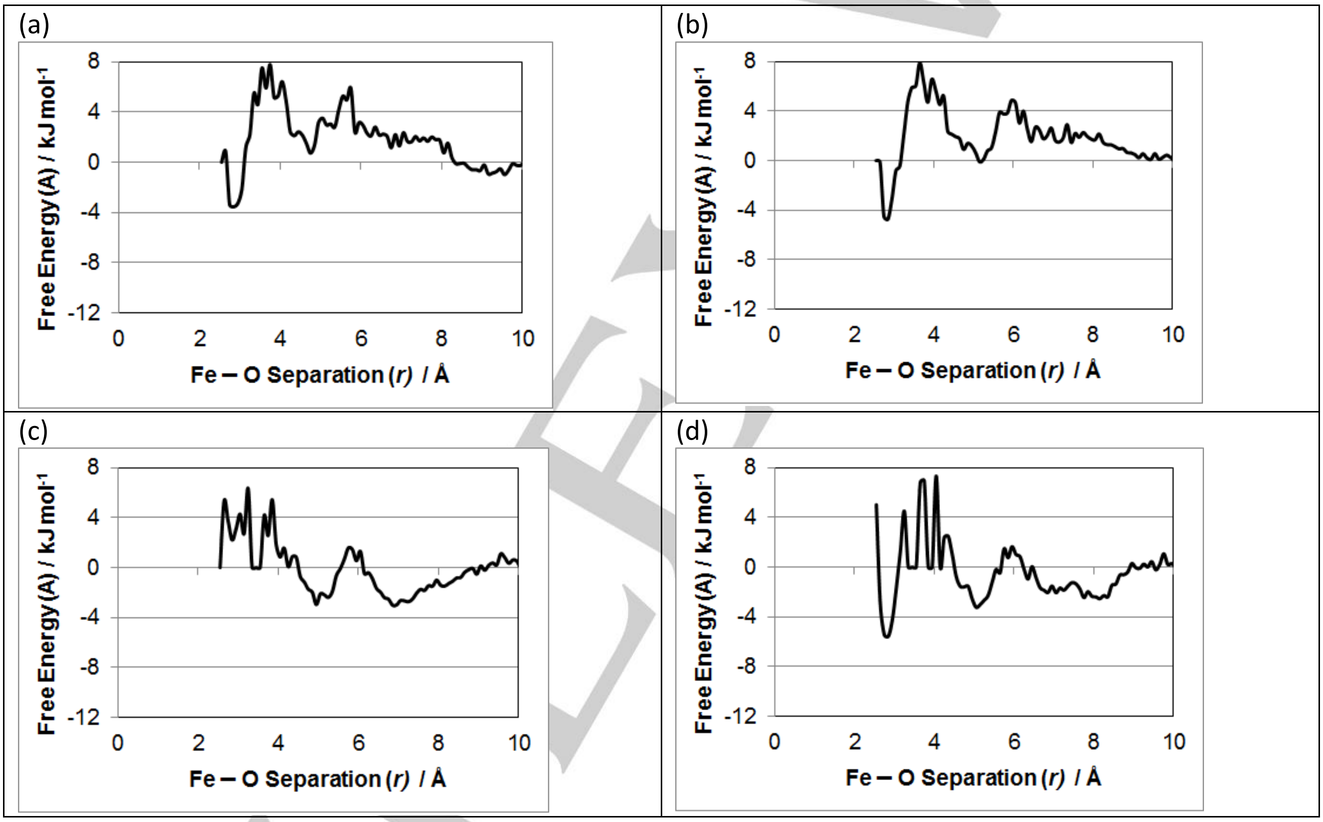

Figure S8: Fe--O free energy profiles at 300 K for the sites where only limited binding is observed in the MD simulation: a) and b) the oxygen atoms in GLU E12 and c) and d) the oxygen atoms in the ASP group at E11.

Supplementary Methods

Electron Microscopy (Figure S2)

The products from an X=0.3 or X=0.6 co-precipitation reaction were first dispersed in water, dropcast, and dried onto carbon coated copper TEM grids (Agar Scientific, UK) under a gentle stream of nitrogen. Electron microscopy was performed on a Technai G2 Biotwin 120kV operating at 80kV equipped with a wide angle Gatan MS600CW camera or on a Philips CM200 operating at 100kV. At least 10 images, distributed around the TEM grid, were obtained.

Small scale magnetic analysis (Figure S3)

For small scale precipitation experiments a 50 mM solution in deoxygenated ultrapure water was prepared of both $Fe(SO_4)$ and $Fe_2(SO_4)_3$. Appropriate volumes of each solution were mixed in vials to generate ratios of Fe^{3+} to total iron (X) of 0.2, 0.3, 0.4, and 0.5, in a final volume of 250 µl. Identical mixtures were prepared with the addition of 10 µg of purified Mms6. 22 µl of deoxygenated 1 M NaOH (R = 1.76) was then added sequentially to each vial before sealing. The magnetic susceptibility of each sample was measured using an MS2G single frequency sample sensor (Bartington instruments Ltd). Prior to each reading being taken the precipitate which had formed was dispersed in solution by brief mixing. Readings were taken after 30 minutes. The difference in magnetic susceptibility between the control and Mms6 supplemented sample was calculated and averaged over three replicates for each precipitation condition to generate a mean fractional percentage difference between the control and Mms6 supplemented sampels at each X value to directly compare with the values obtained from the mass balance diagram.

Supplementary Tables

Reaction (X)	E_1	E_2
0.2 Mms6	0.57	2.13
0.2	0.56	2.02
0.3 Mms6	0.84	2.13
0.3	0.86	2.10
0.4 Mms6	1.14	2.32
0.4	1.15	2.34
0.5 Mms6	1.39	2.38
0.5	1.40	2.44
0.6 Mms6	1.63	2.56
0.6	1.62	2.57
0.72 Mms6	1.95	2.77
0.72	1.91	2.74

Table S1. Equivalence points E_1 and E_2 for reactions at different values of X either with or without addition of Mms6. (Data used to produce Figure 3b)

Reaction (X)	Ferrous hydroxide (%)	Green rust (%)	Magnetite (%)
0.2 Mms6	55	25	20
0.2	45	52	3
0.3 Mms6	27	51	22
0.3	27	60	14
0.4 Mms6	27	26	48
0.4	34	18	50
0.5 Mms6	9	37	57
0.5	18	18	65
0.6 Mms6	3	15	83
0.6	7	8	85

Table S2: Relative percentages of each iron species at E_2 calculated from the mass balance diagram. (Data used to produce Figure 4b). All values rounded to the whole numbers.

WILEY-VCH

Reaction (X)	Mean magnetic moment µ (cgm, a.u)	Standard deviation (cgm, a.u.)	% difference (+Mms6 vs control) in µ	Standard deviation	% difference in quantity of Fe_3O_4 (from Table S2)
0.2 Mms6	0.16	0.03	53.3	43.8	85
0.2	0.08	0.048			
0.3 Mms6	0.82	0.43	20.9	16.0	30
0.3	0.65	0.157			
0.4 Mms6	1.13	0.21	-20.4	9.7	-4
0.4	1.36	0.394			
0.5 Mms6	1.48	0.412	10.6	10.0	-12
0.5	1.3	0.032			

Table S3. Magnetic measurements of precipitated samples with R = 1.76 of NaOH added (column 1 and 2). Columns 3 and 4 show the fractional % difference and the last column gives the values in this form obtained from Figure 4B and table S2. (note values of µ in cgm (arbitary units), Fractional % difference have no units)

WILEY-VCH

Table S4. Chemical shift assignments for C20Mms6.

	Predicted random coil shifts					Observed shifts					Difference between observed and random coil shifts						
Residue	HN	HA	HB1	HB2	HG	HN	HA	HB1	HB2	HG	HN	HA	HB1	HB2	HG	SC mean*	Corrected shift**
K1	8.392	4.351	1.776	1.747	1.359	8.317	4.322	1.840	1.749	1.462	0.075	0.029	-0.064	-0.002	-0.103	-0.056	0.001
S2	8.343	4.463	3.873	3.847		8.457	4.447	3.894	3.894		-0.114	0.016	-0.021	-0.047		-0.034	0.023
R3	8.422	4.360	1.793	1.762	1.554	8.451	4.345	1.885	1.778	1.653	-0.029	0.015	-0.092	-0.016	-0.099	-0.069	-0.012
D4	8.307	4.628	2.716	2.664		8.400	4.605	2.748	2.618		-0.093	0.023	-0.032	0.046		0.007	0.064
I5	8.140	4.142	1.783			8.058	4.164	1.907			0.082	-0.022	-0.124			-0.124	-0.067
E6	8.430	4.342	2.022	1.997	2.258	8.482	4.311	2.094	1.986	2.319	-0.052	0.031	-0.072	0.011	-0.061	-0.041	0.016
S7	8.384	4.425	3.873	3.847		8.237	4.440	3.888			0.147	-0.015	-0.015			-0.015	0.042
A8	8.345	4.351	1.357			8.337	4.356	1.416			0.008	-0.005	-0.059			-0.059	-0.002
Q9	8.248	4.39	2.046	2.015	2.302	8.365	4.373	2.139	2.009	2.393	-0.117	0.017	-0.093	0.006	-0.091	-0.059	-0.002
S10	8.371	4.451	3.873	3.847		8.401	4.480	3.967	3.877		-0.030	-0.029	-0.094	-0.03		-0.062	-0.005
D11	8.414	4.644	2.716	2.664		8.497	4.627	2.731	2.731		-0.083	0.017	-0.015	-0.067		-0.041	0.016
E12	8.233	4.264	2.022	1.997	2.258	8.349	4.249	1.998	1.998	2.319	-0.116	0.015	0.024	-0.001	-0.061	-0.013	0.044
E13	8.401	4.307	2.022	1.997	2.258	8.331	4.255	2.048	2.048	2.430	0.070	0.052	-0.026	-0.051	-0.172	-0.083	-0.026
V14	8.216	4.070	1.985		0.816	8.074	3.978	2.099		0.954	0.142	0.092	-0.114		-0.138	-0.126	-0.069
E15	8.554	4.290	2.022	1.997	2.258	8.385	4.260	2.009	2.009	2.309	0.169	0.030	0.013	-0.012	-0.051	-0.017	0.040
L16	8.324	4.350	1.612	1.521		8.228	4.328	1.693	1.608		0.096	0.022	-0.081	-0.087		-0.084	-0.027
R17	8.350	4.337	1.793	1.762		8.224	4.277	1.857	1.828		0.126	0.060	-0.064	-0.066		-0.065	-0.008
D18	8.357	4.570	2.716	2.664		8.333	4.565	2.731	2.663		0.024	0.005	-0.015	0.001		-0.007	0.050
A19	8.253	4.309	1.357			8.200	4.272	1.433			0.053	0.037	-0.076			-0.076	-0.019
L20	8.131	4.304	1.612	1.521		8.140	4.277	1.755	1.614		-0.009	0.027	-0.143	-0.093		-0.118	-0.061

*Side chain mean. Difference in side chain shifts averaged over all side chain positions within the particular residue.
**Corrected shift is the difference between the “SC mean” and the mean “SC mean” which is averaged over all 20 residues within the peptide (mean SC mean = -0.057).

WILEY-VCH
